# Strategic management as adaptation to changes in the ecosystems of public hospitals in Israel

**DOI:** 10.1186/s13584-020-00424-y

**Published:** 2020-11-24

**Authors:** Lior Naamati Schneider

**Affiliations:** Hadassah Academic College Jerusalem, Department of Service Organization Management, Health Track, Jerusalem, Israel

**Keywords:** Hospital management, Health system management, Strategic health management, Health system in Israel

## Abstract

**Background:**

Health systems worldwide function in constantly changing local and global ecosystems. This is the result of economic, demographic, and technological changes, among others. In recent decades Israel has started implementing reforms in the public health services that have led to far-reaching changes in the health system, and consequently, increased competition within it. The impact of these changes has been exacerbated by pressure to reduce per capita public health costs, coupled with increased demand and greater health awareness. All these changes have created a turbulent environment for healthcare organizations in Israel. To cope with this dynamic environment, various parts of the system have had to adopt appropriate management behaviors and business styles. This study, carried out in six public hospitals in Israel, evaluates the nature and degree of adaptation, implementation, and inculcation of management strategies in public hospitals in Israel, using the Ginter model of strategic management of health organizations.

**Methods:**

The study used semi-structured in-depth interviews of key figures in the health system and managers at various levels in the hospitals and HMOs included in the sample. The 55 interviews, conducted in two time periods, were analyzed in accordance with an established theory of qualitative methodological analysis.

**Results:**

The main findings are that the health market and hospitals in Israel are increasingly adopting competitive business behaviors. But strategic managerial behavior has been adopted only in part, and there is a lack of collaboration between staff and management in defining goals and strategic activity. These are obstacles to change and inculcation of the strategy in hospitals.

**Conclusions:**

This study affords an important view over time and a better understanding of the behavior and adaptation of hospitals in Israel to their constantly changing surroundings. Adapting and inculcating appropriate managerial strategies in hospitals requires close collaboration between staff and management; its absence is an obstacle that contributes to partial, and possibly counter-productive, strategic behavior.

The solution may lie in a combination of changes: providing hospital management with the necessary tools and broad professional support by the Ministry of Health; organizational changes in hospital management and departments; the creation of a clinical leadership role; and a self-supervised planning system .

**Policy recommendations:**

These recommendations regarding training and the direction and organization of the change, coupled with systemic oversight of them by the Ministry of Health, will enable the system to become more efficient. They are particularly relevant today because the Covid-19 pandemic has exacerbated and highlighted Israeli public hospitals’ financial and organizational problems. Hospitals that already faced many challenges have had to cope with an unfamiliar medical crisis and a reduction of elective medical activity, causing them various types of damage, especially in term of economic stability.

The hospitals’ fragile situation must become a top government priority because it can no longer be ignored. To achieve a strong healthcare system with stable hospitals, able to respond both to everyday challenges and to crises like the current pandemic, policymakers must provide financial and organizational support alongside managerial training, while maintaining an overall systemic plan.

## Background

Health systems worldwide function in turbulent and ever-changing local and global ecosystems. This complex environment contains a multitude of factors—demographic, economic, political, and legislative—as well as technological developments, changes in lifestyle, and social influences [1]. Health organizations must also contend with a rich ecosystem of many stakeholders, such as policy makers who grant legitimacy, funders, the public, competitors, employees, and managers.

The constant changes in these determinants in recent decades have created complex ecosystems with changing needs, exacerbated by the added pressure to reduce public expenditure on health [2–4]. This local and global ecosystem creates a dynamic, unstable, and competitive environment that affects the provision and management of health services [5].

In addition, all countries must balance the right to health with the extent of involvement it requires. Moreover, making equality a priority requires oversight and regulation [6], raising many administrative and financial problems for health organizations in a competitive, regulated financial market: They must walk a tightrope between financial business autonomy and close regulatory oversight [7].

This study evaluates the nature and degree of adaptation, implementation, and inculcation of management strategies in public hospitals in Israel.

### The local ecosystem—the health system in Israel

In considering the factors that make up the ecosystems that affect the Israeli health system, one must take into account not only the global changes in recent decades but also the structure of the local health system: Because of its brief but complex history, it is a patchwork of diverse bodies with complex relations among them and various types of ownership. Every Israeli citizen is entitled to health coverage by one of four nonprofit HMOs that compete with each other. The HMOs use various payment systems to purchase hospitalization services from the hospitals. The distribution of hospital beds, in terms of ownership, location, and main costs—such as purchase of expensive equipment—is arranged and overseen by the Ministry of Health [5, 7]. The public system comprises hospitals with various types of owners—including nonprofit organizations, HMOs, and the Ministry of Health—and competes with a private system [7]. This pluralism generates unique organizational problems. An example is the multiplicity of roles of the Ministry of Health: The legislator is also the owner and regulator of some of the hospitals [8]. Another example includes the changes and reforms of the public health services, whose crowning achievement was legislation of the National Health Insurance Law, 1995, which was to have led to a reorganization of the Ministry of Health and its involvement in the direct operation of health organizations. This reform, implemented only in part, led to far-reaching changes in the health system whose ramifications were manifested in the behavior of the health organizations [9, 10].

For the hospitals, the ramifications were indirect but significant from the outset. The new financial arrangements, capitation arrangements, updating of fees, and competition among the HMOs generated pressure on the hospitals and a decline in the HMOs’ use of their services. This generated an acute need for the hospitals to develop new services and compete with each other [5].

This need, in turn, is forcing them and their managers to adopt business-oriented modes of behavior, so as to adapt continuously to changing market conditions, stiff competition, and conditions of uncertainty [2, 3, 11], and creeping corporatization has intensified in the hospitals, forcing them to operate in accordance with a business plan [12].

To survive, public hospitals must balance their books and generate income beyond their usual funding. Among the creative business solutions has been the establishment of research funds associated with the hospitals. Under a 2002 law, they function as legally separate entities and make possible activity outside regular work hours, research, employment, medical tourism, and other revenue-generating business activity [5, 7]. The funds provide administrative autonomy and flexibility in the use of resources and employment. They are subject to regulation, but they enjoy broader freedom of activity than do the hospitals. Today, about one-third of all the government hospitals’ activity is conducted through these funds, which act as separate economic units (apart from 20% overhead paid to the hospitals). They contribute substantially to shortening the wait for hospitalization and surgery, employment of senior doctors, and medical tourism as well as to the hospitals’ positioning, reputation, construction of added value, attractiveness, and financial stability. How the funds are managed varies from hospital to hospital and leads to far-reaching differences in the hospitals’ turnover, in accordance with their size, geographic location, and services provided. (Whereas, for example, in 2018 Sheba Hospital reported a turnover of NIS 965.3 million, that year Poriya Hospital reported NIS 52.2 million) [13]. The corporations, the introduction of private services in some of the public hospitals (Sharap), and the broad range of commercial services provided in conjunction with the hospitals constitute hybrid business and organizational solutions in which there is a blurring of the boundaries between the types of organizations and their aims [7].

This behavior was addressed in the State Comptroller’s Reports of 2008 and 2015, which pointed out that to some extent creeping corporatization was manifested in the activity of health corporations and public hospitals. Such hybrid organizations and such behavior generate a problematic, inherent lack of global oversight of budgets and management [14].

The changes in the overall environment of hospitals are lasting, and they require hospitals to change extensively and permanently. Recent years have seen a continuing crisis caused by the unequal encounter between the health system’s ability to provide high-level health services and the state’s limited ability to fund these services for the entire population. Many factors—the global ecosystem, the changes in the local ecosystem described above, budgetary constraints, a substantial increase in the use of medical services, new financial arrangements with the HBOs and hospitals, updating of fees, competition by the HMOs, and the development of private supplementary insurance policies—have all combined to undermine the sought-after financial stability and generate a chain reaction of limiting the use of hospital services by the HMOs while creating great competition among the hospitals. This has resulted in continuous financial pressure, great work pressure, and great uncertainty for the hospitals [5]. Having to function with budgetary shortfalls is driving an increase in the business behavior of hospitals and a blurring of boundaries between being regulated public organizations and organizations acting in an autonomous business manner with the aim of trying to survive or at least achieve financial stability [7].

All the above factors make it difficult for the health system to provide quality health care for all the state’s citizens, thus threatening the collapse of Israel’s public health system [15]. To cope with this situation, changes have been made in the structure of payments to the hospitals for their services, in the mutual relations between various stakeholders in the health system [16], and in the positioning and differentiation strategy of hospitals in relation to their competitors [17]. The result is that hospitals are becoming more independent and must become more competitive [18].

In many respects, the health services, and especially hospitals, act in a manner very similar to that of business organizations [19], even though they are not independent entities and are required to provide service for all regardless of profitability considerations. However, despite the public hospitals’ resemblance to business organizations, there are many differences: in hospitals’ ability to make decisions independently, in their limited financial behavior because of their limited income, and in the externally determined limitations on contracts and staff positions. There are also organizational differences, such as financial and managerial independence of the organization’s units—the clinical departments, for example [4, 20]. Nevertheless, the comparison between hospitals and purely business organizations, despite its inherent problems, is necessary for the hospitals’ survival [21], although, while analyzing their adaptation to the changing ecosystems, we must recognize that making such a shift—including changes in the approach and in the manner of providing services, as well as changes in management of the human capital, with an emphasis on the degree of profitability and utility as core goals—involves a complex and difficult process of organizational change [22].

Many studies have addressed the complexity of medical–managerial relations in health organizations, particularly in hospitals [23, 24] which makes organizational change and adaptation to change more difficult and charged. The difficulties include the inherent conflicts of interest and power relations within the organization [25]. Whereas hospital managers must address the financial aspects and adopt a global view of the organization, the doctors are concerned with treating patients and are supposed to ignore considerations of cost and benefit. This conflict [26] is combined with the inherent managerial tensions related to work conditions, wage agreements, and promotion [26]. In Israeli hospitals most of the managers are doctors by profession, but they are not currently treating patients. Department heads, however, are senior doctors and must be physicians and managers simultaneously [26]. The departments are organizational units in which the in-house treatment of patients takes place. The department heads fulfill their managerial role—for example, obtaining equipment and human resources—in addition to treating patients and being responsible for the department and treatment in it. The departments are not managed as closed financial units, though often their activity is measured by their degree of profitability [27], and their heads do not have full autonomy in decision making. In some cases departmental budgets have been implemented but do not include personnel costs. The degree of autonomy in managerial and financial decisions, including those regarding the departmental budget, varies from one hospital to another in Israel and this, too, constitutes grounds for tension in the doctor–manager relationship [7].

In Israel this conflict combines with strained work relations resulting from labor disputes and a lack of trust and transparency vis-à-vis the Ministry of Finance and even vis-à-vis senior doctors of health organizations and hospitals. This complexity of relationships is another difference between purely business organizations and health organizations and increases the difficulty of inculcating change and adaptation to a dynamic reality. It is particularly important in hospitals that must adapt very rapidly to reduce the negative effects on the services provided.

These changes require adaptation at all levels of the organization and in the managerial and therapeutic staff [21]. Two levels of obstacles hamper the inculcation of change: the external context (the array of factors in the environment, under-budgeting, and a problematic distribution of resources) and the internal context (the array of forces within the organization, the power relations and tensions at the various levels of management, the doctor–manager relationship, the organizational culture, problems in the organization’s structure, and the granting of managerial and budgetary authority to the doctors who head the departments).

The premise is that to succeed in today’s market, hospitals must adopt a managerial approach and invest in strategic planning to overcome the obstacles in their institutional and operational structure, described in this section, and to achieve financial and managerial efficiency.

### A model of strategic management

In this study, the analysis of the strategic behavior of hospitals is based on Ginter’s model [19] of strategic management in health organizations. According to this model, strategic management includes three stages: strategic thinking, strategic planning, and strategic momentum.

All levels of the organization, and not just the management, must pass through all three stages. This generates a critical structured process at every level, in which decisions are made in a manner that is suited to the changes and the adaptation to them. The processes are continuous, flowing back and forth in many ways between the stages, but are led from above and require adaptation at every level of the hospital [19].

#### The strategic thinking stage

The initial stage involves an analysis of the external changes and recognition of the organization’s need to change so as to adapt to the external changes in an optimal manner. This stage requires a broad view of the conditions of the environment [1], involving observation and review of the past and the present and creation of a window that looks to the future. It requires a holistic view of the surrounding situation (both internal and external) and of the changes, as well as an analysis of the ramifications of these changes. In many senses, strategic thinking requires elements of leadership, a collection of characteristics and actions, and not only status and position in the organization [28, 29].

#### The strategic planning stage

In the second stage, strategic thinking is translated into a plan of action, a sequence of steps the organization must take in order to implement its mission and vision and achieve its goals [19, 21]. This stage involves data collection and analysis, brainstorming, group thinking, establishment of emphases and organizational goals, organizational focus on decision-making processes, and construction and documentation of action plans, from the general to the particular, in various time frames. The product of this stage is an effective action plan consisting of goals and details of how each unit in the organization will contribute to the strategy, distribution of roles and tasks with a time table, and resources for carrying out the tasks [19]. It is essential that this stage be carried out by many staff members or a large number of key staffers with different perspectives to ensure creative and critical thinking regarding solutions [30].

#### The strategic momentum stage

This stage is crucial for carrying out the plans of the preceding stage. By attempting to coordinate the organization’s internal state (including culture, structure, resources, and services) with its external environment (political, regulatory, economic, technological, social, and competitive forces), this stage ensures continued implementation of the strategic plan and includes a continuous process of evaluation of the environment and the organization. This stage is part of the inculcation of strategic thinking in the organizational culture and philosophy, and as such it is directed at both managers and staff. It is a continuous learning process in which the changes are inculcated in accordance with the preceding stages of thinking and planning. Those changes and their degree of success, after being evaluated on the basis of data, are abandoned or continue to be developed. At this stage it is very important to commit all levels of management and staff to the organization’s strategic goals, mission, and vision, while adapting permanent and defined advance planning to changing market conditions, which require constant flexibility and creativity [19].

In implementing the three stages of strategic management, the organization must emphasize balancing efficiency with the usual work routines and adaptation to frequent changes [19]. In such a dynamic and changing environment, it is difficult to develop a continuous competitive advantage ([19, 31]. However, use of strategic management can be advantageous in positioning and competing amid changing conditions. As part of strategic management, it is important also to change the organization’s internal environment as changes occur in the external environment. Such internal changes are accomplished by developing an internal policy that espouses change, the establishment of communication, and the organizational culture, and by empowering the people who adopt the changes [21].

The current study uses Ginter’s model [19] to evaluate the degree of strategic planning in Israeli public hospitals and how and to what extent the managers and the doctors have coped with the transformation of their organizations into business-oriented entities.

## Methods

This study used a qualitative analysis based on grounded theory [32, 33]. This method was selected because the strategic changes in the hospitals were implemented in a complex situation subject to many environmental influences, which, for the most part, were mediated by individuals within the hospital. Therefore, knowledge of their character and worldview are crucial to understanding the processes of change and how they were led. In the attempt to understand fully how hospital managers and employees coped, they were given an opportunity to express their experience in personal terms that cannot be quantified and can be analyzed only qualitatively.

### Participants

Fifty-five interviews were conducted with key figures and senior staff of hospitals, HMOs, the Ministry of Health, and the Ministry of Finance. Six of the large hospitals in Israel were selected for the study on the basis of their size, location, number of beds, and ownership. Several levels of hospital management were studied: the top managers and their assistant managers, the marketing managers, the public relations officer, and heads of the cardiac surgery, gynecology and obstetrics, and children’s departments. These types of departments were selected because they have relatively broad strategic activity.

In addition, the directors of clinics and districts of the HMOs (some of whom also held positions in hospitals) and senior managers in the Ministry of Health were interviewed as part of the mapping of views and attitudes toward general strategic topics.

Several interviews with senior doctors and policy makers in the health system, conducted in the media between 2012 and 2019, were included in the data. Also, various hospital documents were used, including hospital bulletins, statistical information about the hospital, and hospital marketing brochures, as well as reports in the media and on official hospital websites. All these served as background for the interviews and the analysis.

### Conduct of the study

The participants signed a consent form before the beginning of the interview, following assurance that both they and their institution would remain anonymous and with the understanding that they could stop the interview at any point. The interviews were recorded with the interviewees’ permission and transcribed close to the time of the interview, in accordance with transcription rules.

### The interviews

The data were gathered by means of in-depth, semi-structured interviews that were conducted in the participant’s workplace. Such interviews are characterized by flexibility in the interview structure, many open questions, and encouragement of the interviewee to tell his or her story. The interviews were conducted in two time periods—between 2010 and 2013, as part of a study on the topic, and between 2018 and 2020, to enable the examination of changes in perceptions and actions over time.

A page of general questions was prepared in advance. It was not shown to the interviewee but constituted the basis for a conversation with the interviewee. First, we examined the interviewees’ perspective on the changes in the behavior of hospitals and the health system in general in recent years as a consequence of the reform of the health system and the National Health Law. Then the interviewees were asked to relate to changes their hospital had undergone in light of the reform of the health market. The conversations focused on topics relevant to the study: long-term strategy, how it was conducted, the impact of budgeting changes on the management of hospitals, their marketing activities, and competition between them.

The data gathered underwent a thematic analysis that generated initial categories. This was followed by a search for main topics and themes and an analysis of them. At a later stage, observation and mapping of the array of themes, sets of relations, and connections between various levels of the existing topics enabled the researcher to define a map of concepts and connections that creates a more comprehensive picture of the data gathered [32–34].

## Results

The full qualitative thematic analysis of the interviews is not presented here, because the full findings go beyond the scope of this article.

This section focuses on the parts of the interviews that are relevant to the substantial changes with which Israeli hospitals have had to contend in the last two decades. The aim is to examine the hospitals’ strategic management following the opening of the health services market to competition and the hospitals’ behavior as business entities.

Analysis of the data reveals agreement regarding competition: All the participants mentioned that there is great competition between the hospitals in a given region as well as competition between hospitals in different parts of the country.

*[…*] *a very competitive market […] [the competitors] are all of them, all of them, all the leading hospitals in the country […*].

*[…] It’s us against [name of hospital] […*]; *there is competition between us and [name of hospital]; there is competition in all aspects.*

From the analysis of the attitude toward transforming the hospital into a business organization it appears that most of the interviewees saw this characteristic as a dominant factor that influenced the hospital’s behavior, both on the medical and academic levels and on the financial level.

*… The hospital became a financial system that needs to work according to rules of the financial system, and this is the most significant change resulting from the reform.*

*Hospitals today are managed, actually, as corporations, even though they are not corporations.*

There is agreement that the main defined purpose of each hospital, as in for-profit businesses, is to turn a profit or at least to break even.

*The business agenda, which people at the top continue to set, is that the hospital’s interests must be financially balanced and successful.*

Nevertheless, several topics arose that pointed to the inherent dissonance in the linkage of business agendas and strategies with medicine in the hospitals and the health systems in general. Among the interviewees there was no clear support for the supremacy of finances; sometimes, for reasons of prestige, they preferred popularity and high volume of activity over profitability.

*Hospitals must have financial thinking […] but only soft financial thinking; money should not drive the entire system, and we are going in that direction.*

*[…] In principle, we are doctors first of all … We are not business managers. That means that first of all we desire medical excellence […].*

One of the ways that hospitals have tried to improve their financial situation is to increase the efficacy of their management.

*[…] There are great changes in the behavior of our doctors […] Oversight systems have been created in the departments, […] all kinds of ways to examine the department’s activity.*

Another way is to make the hospital’s units more independent financially and managerially, as in business organizations. From the interviews it appears that this initiative was implemented only in part, both because of objections raised by department heads and because of existing limitations.

*[…] Our financial and managerial independence is very limited […]. Our decisions […] are meaningless […]. We have no budget. That means that my ability to understand the business of medicine is limited […]. (department head).*

According to department heads, the problem with this initiative is that they do not have the knowledge or the tools to manage their departments by themselves and thus their units would collapse very quickly.

*Let’s say that tomorrow they make this unit independent financially. I will fall apart in a very short time.*

As part of the move toward business-oriented behavior and balanced books, hospitals have introduced strategic goals and aims, including increasing profits by increasing the volume of patients.

*There is pressure from management for full capacity, that there be more patients in the hospital.*

The hospitals are also transforming the patients into customers and trying to become more patient oriented. For example, hospital staffers have been trained in customer service and to treat patients in a more personalized manner, because patients are more aware of their rights and have learned to expect better service.

*We are doing this today in a very aggressive, intensive manner—workshops that focus on appropriate sets of personal relationships, good service, and […] how you relate to patients.*

The existence of comprehensive business strategies in the hospital arose only indirectly in the interviews. Most of the participants pointed out that there is a business agenda or a particular strategy, and even said that it is felt in the hospital. Nevertheless, the interviewees (especially at the level of department heads) were not always able to describe it in detail; they merely pointed out that such a direction exists. They related only to the significance of the business strategy for them: a general increase in the volume of activity. As most of them saw it, the hospital’s business philosophy focused on an increase in profitability, but the strategic means for achieving the goals were solely the domain of management.

*[…] Generally, the professional echelon knows how to market itself as a good doctor; it doesn’t know how to talk about strategy; the word “strategy” in medicine is very, very hard. The doctors are programmed, and the nurses even more so, to work one-on-one. In one-on-one there is no strategy. There are only trees, no forests.*

Clearly, the inculcation of the strategy is not uniform. All of management talks about strategy and inculcating it (even when the nature of the strategy is not necessarily clear). Often, the strategy is imposed from above, although sometimes the department heads point out that they are the initiators of most of the actions, so that in actuality the direction of inculcation is reversed. Either way, the department heads are not always involved in the decisions and are not even always aware of all the relevant data.

*Sometimes there are initiatives that come from below, but a large portion of them come from above; it’s a combination. It depends on what it’s about. Things that have a lot of regulation and great monetary potential are mostly from above […].*

*There is a business agenda. There are people who broadcast this from above all the time. This hospital has a huge interest in being financially balanced and economically successful […] and this trickles down …*. I*t’s definitely there.*

Department heads sometimes feel cut off from the managerial echelon, both at the level of strategic planning and at the level of general knowledge. Many of them pointed out that if they do get a report from management, usually it is only about income, and that there is no collaboration at the planning level. Some of the department heads are content with this situation and are not interested in changing it. But others point out that it is problematic in terms of efficiency of the system and management of the department. It is evident that often, even within the same hospital, the heads of different departments have different approaches regarding the degree of collaboration and independence they would like to achieve.

*[…] Our goal is to be a better department than the others; to be better professionally, medically. [For] management—what counts is the bottom line, then it goes into the topic of business competition[…]. [That’s] not for us […].*

*I can talk to you a lot about business, but it’s just lip service; it doesn’t happen here. […] The work of the department head in general doesn’t have a business component.*

## Discussion

The following is an examination of the findings in light of Ginter’s model of strategic management. As noted, the model’s first stage is strategic thinking, which includes an analysis and recognition of the required change and its necessity for achieving the organization’s optimal adaptation to its environment. This stage requires a broad view of the surrounding conditions and the changes and an analysis of the ramifications of these changes [19].

From the statements quoted above it appears that the interviewees were aware of the local and global changes that affect the health market in general and hospitals in particular. Moreover, they declared the immediate need for changes in the hospital in order to adapt to these external changes. However, they did not demonstrate an organized thought process in which comprehensive information about the levels of the various changes in the environment were examined and on the basis of which events and scenarios for achieving the optimal adaptation were built. There was a lack of clarity regarding the main goals and their necessity as part of a comprehensive strategic master plan. This indicates only partial fulfillment of the model at this stage.

A decisive part of this stage is collaboration and encouragement of all the organization’s members to participate in the process of strategic thinking [19]. However, the findings show a distancing and a disconnect between the hospital’s management and the department heads. There is informal agreement regarding the various motives and interests underlying the decision making and this includes a perception that strategic thinking and looking at the big picture are the exclusive domain of senior management.

*Why do I need the doctor to mess with hospital strategy?* (*hospital manager).*

This attitude is contrary to the model, which encourages collaboration and transparency at all levels of the organization, and it constitutes support for earlier studies on transparency and collaboration in doctor–manager relations, according to which the level of these elements in health organizations is lower than in business organizations [35].

In the model’s second stage—strategic planning—strategic thinking is translated into an action plan, a series of steps the organization must carry out [19, 21]. The products of this stage are supposed to include goals and details of each unit’s contribution to the strategy [19]. It is crucial that this stage be carried out by many staff members, including key personnel with differing views, to ensure creative and critical thinking for finding solutions [30].

Most of the interviewees stated that their hospital had an agenda and/or a particular business strategy that was the basis for action. Nevertheless, regarding the two main goals that they named—improving the hospital’s financial situation and increasing the volume of patients—they could not point to a detailed action plan for either the short term or the long term that laid out the steps that needed to be taken. Alternatively, they stated that they did not have access to such a plan or were not exposed to it. Consequently, this stage of the model, too, is executed only partly, and there is great variability in the adoption and exposure to it at the various levels of management.

The final stage of the model is the strategic momentum, the execution of the plans formulated in the preceding stage. At this final stage, the changes in the organizational culture are meant to be inculcated at all levels of management and staff [19].

When the statements of hospital directors are compared to those of department heads, it appears that there is a clear disconnect between the two and sometimes even mutual rancor over the fact that they have not worked together in accordance with the model.

*I can tell you that most of the department heads have no idea … about these matters … (hospital director).*

*Our financial independence is very limited … I don’t have a budget … I get no feedback … so I don’t even know which activity is profitable and which is not, or how we should cut costs and expenses […]. I know that some of those reports exist somewhere. (department head).*

At the momentum stage, the organization is supposed to continue implementing the action plan by attempting to coordinate its internal state (that is, resources, services provided, structure, policy) with the changing external environment. This process of evaluating threats and potential opportunities in the environment and ensuring that the hospital is better adapted to it is crucial for its success in a competitive market [19]. Given the complexity of the Israeli health market and the hospitals’ inability to act as strictly business organizations, it appears that despite their ability to evaluate the external environment, they have limited ability to adapt their intra-organizational state fully. Decision making and setting short-term and long-term goals based solely on maximization of profits and reduction of costs is impossible for them, for they must take other factors into account, such as the Ministry of Health and the hospital’s stakeholders.

In summary, the findings reveal that there is a strategic business basis for the interviewees’ activity, although not all of them were able to describe it in detail. Two main goals that were named were increasing profits and increasing the patient volume. With incorrect planning and management, these goals could conflict with each other.

Most of the interviewees’ statements dealt to some extent with strategic thinking, but they were sporadic and not organized. For the most part, they did not dwell on strategic planning or strategic momentum. It appears that all the players are aware of the external changes and the need for rapid adaptation to them. However, the stages of strategic thinking and planning, even where they exist almost in full in the management, are barely apparent at other levels of the organization. There is a great gap between the behavior of the hospitals’ management and that of various departments. Staffers and department heads are not involved in the initial strategic planning stages, making the entire process largely irrelevant to them and creating a barrier between them and management. This “division of labor” undermines strategic planning and prevents collaboration of everyone involved. The absence of collaboration and inculcation of formulated plans sometimes leads to the failure of strategies for change. This is one of the factors underlying the hospitals’ problematic financial behavior and the limited impact on their income, resulting in large deficits.

*As of today, not a single general hospital in Israel is able to live on the Ministry of Health budget […].*

*The public hospital in Israel, as in the rest of the Western world, is not viable […]. True public medicine in a socialist country that wants to provide treatment but not profit is not viable.*

These findings are supported by earlier findings that show that the financial stability of public hospitals in Israel is constantly being undermined [15, 17].

According to the findings in this section and the previous one, the examination of the implementation of Ginter’s strategic model in hospitals shows that despite broad business activity, strategic activity is inculcated only partly. The organizational difficulty is manifested in tense and complex doctor–manager relations, conflicts of interest, and budgetary pressures. These factors, combined with external financial and political difficulties, hamper management in inculcating comprehensive organizational change and broad business strategies. Consequently, the hospitals behave in financial survival mode—introducing individual, primarily short-term, initiatives using the available resources, with no overarching plan and no sufficiently strategic managerial behavior—to maximize activity and make it more efficient [15].

## Conclusions

The environment of hospitals is changing constantly, and public hospitals are increasingly adopting business-oriented behaviors to survive. These findings are similar to earlier findings in studies of competition between hospitals in Israel [36, 37] and even create a research continuum with the interviews conducted and analyzed in other studies [4].

Hospital directors are becoming increasingly focused on strategy and goals, and financial considerations are increasingly driving their behavior. However, these changes are not taking place in the same way at other levels of management, that is, among department heads. There is a partial adoption of processes and business strategies in hospitals but also an absence of clear and long-term, multisystemic strategic managerial direction. There is great variability in the approaches toward collaboration and transparency of information and behavior, which impedes inculcation of all the stages of business strategies. According to Ginter’s model [19], it is essential to have full cooperation in the first stage of evaluating the situation and the environment, in setting short-term and long-term goals, and finally in implementing those goals consistently throughout the organization. Although the hospitals are in the midst of the process, it is inculcated only partially, and there appears to be a great gap between the desire for change and the recognition of the need for it and the execution of comprehensive change.

The findings of this study and the data from studies that have analyzed the financial state of the hospitals show that the financial stability of Israel’s public hospitals is constantly on shaky ground [15, 17]. This impels the hospitals to initiate specific actions and strategies without a comprehensive systemic view, potentially leading to waste, duplications, and severe deficits. Coupled with limited resources and constant demographic, technological, and social changes, this situation may lead to a collapse of the country’s public health system [15].

The solution for the health system may lie in providing hospital management with the needed tools and broad professional support by the Ministry of Health for the hospitals’ business-oriented financial behavior. Such support would include the development of professional infrastructure and expansion of the professional knowledge in this field on the part of hospital staff and managers, encouragement of comprehensive managerial study programs, and the introduction of these topics as part of medical practice. Also to be considered is the creation of a self-supervised planning system that acts continuously toward efficiency and affects the setting of financial and medical goals.

As part of this change, it appears that the system must erase the existing boundaries in manager–doctor relations and create a hybrid role that encompasses this problematic duality of roles in a more positive manner: clinical leadership instead of management. The correct combination of these roles and specialized training may contribute to better adaptation to changes and lead to provision of higher-quality services [38] and inculcation of a more successful process of strategic change at all levels of the organization. It is also important to arrange the managerial and financial independence of the departments, with collaboration and transparency, using the medical leadership. Creating managerial flexibility that will enable department heads to participate in the hospital directors’ strategy while maintaining departmental interests will help to empower the staff and make it part of the process of change and adaptation [27].

The dilemma regarding the adoption of business strategies and the degree to which public hospitals are turned into competitive, independent corporations is not unique to Israel. Changes in the global ecosystem that derive, inter alia, from economic, demographic, and technological changes, affect the health systems in many countries. In every country the government has a role in shaping and managing the health system. In England, where the health system faces massive deficits, it is managed and financed by the government, and the level of centralization is greater [39]. Starting in 1989, it allowed autonomous, competitive business activity in the health corporations in a separate entity that is managed and overseen by the government, although in recent years the entity’s freedom of action has been reduced. Estonia, which underwent a dramatic, comprehensive organizational change following its separation from the Soviet Union, has a pluralistic structure in terms of costs, but in that country, too, the hospitals’ managerial and financial functioning is partly autonomous, under supervision and regulation [40]. Similarly, in Norway, which has a lesser degree of centralization, hospitals function autonomously within clear boundaries [41].

It is evident that all the countries using the various models of the tax-funded Western health system are coping with some degree of common financial and managerial challenges and that the direction of change in hospital management is relatively similar in all. The states’ behavior ranges between enabling some degree of autonomous business management in hospitals (“room to maneuver” [7]) and maintaining some degree of centralization and oversight in such management. Inculcating the options outlined above will lead the Israeli market toward more strategic and competitive business behavior like that occurring globally. Direction, training, and organization of the change will enable the system to become more efficient and thus ensure its survival.

### Policy recommendations

Today more than ever before, as Israel contends with the Covid-19 pandemic, the meaning of a strong healthcare system and stable hospitals is comprehensible. In many respects the pandemic has exacerbated the existing problems: Regardless of their difficulties in terms of work pressure, stress, insufficient manpower and number of beds, and financial and organizational problems, hospitals have had to cope with an unknown virus. Moreover, in addition to the stresses engendered by the pandemic, hospitals have suffered a reduction of elective surgeries and other medical activity, harming their economic stability, lengthening queues, causing staff reductions, damaging reputations, and deepening deficits [42].

The exposure to, and awareness of, problems of hospitals and the healthcare system must be channeled make this issue a national priority as part of the overall effort to produce economically and organizationally stable health organizations. Israeli policymakers must generate financial and organizational support along with professional training for public hospitals. They must enlarge budgets and increase the autonomy of hospitals and their departments. Finally, employees must be empowered through transparency and collaboration at all levels of the system to achieve common goals, while maintaining systemic interests and creating strong healthcare system and stable hospitals that can respond quickly to the challenges of ever-changing, unpredictable surroundings.

## Data Availability

The database of this study and the analysis of the data cannot be made public because of the promised confidentiality of the identity of the interviewees and the institutions. Nevertheless, the corresponding author may be contacted for additional information.

## Abbreviation

HMO: Health Maintenance Organization
